# HbA1c and the Severity of Acute Pancreatitis: A Systematic Review and Meta-Analysis

**DOI:** 10.7759/cureus.100125

**Published:** 2025-12-26

**Authors:** Alexander Ainger, Stephen Lam, Bhaskar Kumar

**Affiliations:** 1 Upper Gastrointestinal Surgery, Norwich Medical School, University of East Anglia, Norfolk and Norwich University Hospital NHS Trust, Norwich, GBR

**Keywords:** acute pancreatitis, complications, glycated haemoglobin (hba1c), severity, systematic review and meta analysis

## Abstract

Background: HbA1c, a measure of long-term glycaemic control, has been identified as a potential prognostic risk factor for pancreatitis severity, yet there is a paucity of evidence on its association with pancreatitis outcomes in people with and without diabetes. We, therefore, conducted a systematic review and meta-analysis to assess the current body of evidence.

Methods: Articles from January 1980 to March 2025 were screened using PubMed and the Excerpta Medica database (Embase). Randomised control trials (RCTs), cohort, and case-control studies were permitted for inclusion if they used an appropriate method for both acute pancreatitis (AP) diagnosis and severity classification. Quality assessment was performed using the Newcastle-Ottawa scale (NOS), and random effects models reporting pooled odds ratios (ORs) were estimated in our meta-analyses.

Results: Our search generated 2,270 results, from which two studies were eligible for inclusion with a total of 1,195 participants. Both studies were deemed to be at low risk of bias. The results of our meta-analyses demonstrated an increased odds of developing severe AP with increased HbA1c levels (OR = 2.14, 95% confidence interval (CI) 1.32-3.48). Elevated HbA1c levels were also found to increase the odds for developing local pancreatic complications (OR = 1.71, 95% CI 1.25-2.34) and systemic complications (pooled OR = 2.82, 95% CI 0.49-16.28).

Conclusions: Our review suggests that elevated HbA1c levels may increase the likelihood of developing severe AP as well as local and systemic complications. The results of the review are limited due to the small number of included studies. We recommend that large multicentre cohort studies be conducted to further investigate this relationship.

## Introduction and background

Acute pancreatitis (AP) affects 56 per 100,000 of the UK population annually [1], with 25% of cases classified as severe [1]. Organ failure commonly accompanies severe disease, in which mortality can reach 25% [1]. Management is largely supportive [2], though severe cases may require resection of necrotic pancreatic tissue [3]. Global incidence is on the rise, mainly because of increased rates of gallstone disease in the West [4], but nonetheless, AP remains an under-researched area [5].

Metabolic risk factors are associated with pancreatitis severity [6], with a large multicentre retrospective cohort study of 1,257 participants demonstrating that increasing components of metabolic syndrome correlate with more severe disease outcomes [7]. HbA1c is a form of glycated haemoglobin that reflects average blood glucose over three months [8]. It can be measured at any time without special preparation, making it an ideal measurement of glycaemic control [9]. Although AP is known to predispose patients to diabetes via islet cell destruction [10], the impact of chronic hyperglycaemia on AP development is less well described [11]. Previous studies show a higher incidence of AP in patients with diabetes than in non-diabetic controls [12-13]. One population-based cohort study reported an adjusted hazard ratio (HR) of 1.72 (95% confidence interval (CI), 1.52-1.96) for AP in patients with diabetes, rising to 6.32 (95% CI, 4.54-8.81) in those with prior hyperglycaemic crises [12].

Although the effect of HbA1c on AP has not been fully explored, acute hyperglycaemia and its pathophysiology have been studied. Animal models demonstrate that hyperglycaemia exacerbates inflammation and induces apoptosis in AP [14]. As HbA1c reflects chronic hyperglycaemia, its potential role as a risk factor for AP warrants further investigation.

The aim of this literature review was therefore to assess current evidence on the relationship between HbA1c and AP severity and to determine whether HbA1c should be considered a prognostic risk factor.

## Review

Methods

We conducted a systematic review and meta-analysis of the current evidence on the effect of HbA1c on the severity of AP. The review was designed in accordance with the Cochrane guidelines for conducting and reporting systematic reviews [15]. The protocol was submitted prospectively to the International Prospective Register of Systematic Reviews (PROSPERO; registration ID: CRD42023383895). Data extraction for this systematic review was conducted in accordance with the Preferred Reporting Items for Systematic Reviews and Meta-Analyses (PRISMA) guidelines [16].

Search Strategy

A systematic search of the Medline, PubMed, and Excerpta Medica database (EMBASE) databases was conducted to assess the association between HbA1c and AP. The search strategy was developed with support from a university health sciences librarian to ensure broad and inclusive terminology, incorporating multiple variations of HbA1c (e.g., glycated or glycosylated haemoglobin A1c). Searches were performed using the OVID platform [17] and included literature published up to March 2, 2025. Results were limited to English-language articles, excluding textbooks and case reports (single or multiple). Additional eligible studies were identified through citation screening of included papers. The full search strategy is provided in Appendix A.

Eligibility Criteria

Inclusion criteria: The inclusion criteria were as follows: Studies using a standardised measure of pancreatitis severity, e.g., the Atlanta Classification, or clearly outlined symptoms/outcomes (i.e., end-organ damage, death) so that severity could be determined. Studies that clearly defined the diagnosis of AP using an accepted tool or measure, e.g., computerised tomography (CT) scan or serum amylase. Studies that made a distinction between patients with raised HbA1c with diabetes and those without, to minimise confounding due to diabetic end-organ disease. Studies that focused on diabetic patients exclusively could still be included, however, for separate analysis. Studies where patients who were over the age of 18 or included paediatric populations could be separated from the main analyses.

Exclusion criteria: The exclusion criteria included studies where the focus was on chronic pancreatitis rather than AP or made no distinction in the results between the two, so that the relevant data could not be extracted. Studies examined the effects of hypertriglyceridemia exclusively. Studies examined patients with pancreatic cancer exclusively.

Regarding study inclusion type, we only included randomised control trials (RCTs), cohort and case-control studies for our review, excluding commentaries, case reports, case series, letters, expert opinions and non-comparative studies and reviews. In comparison, the former study types provide a higher quality of evidence [18], the latter being far more prone to bias and tending to feature much smaller population sizes. Any non-English language studies identified were not formally assessed for inclusion, as we had no translation resources.

Outcome Measures

Our primary outcome measure was acute severe pancreatitis. Severity needed to be determined using a standardised method (e.g. Atlanta Classification or Ranson criteria), or clinical measures must have been clearly defined so that severity could be determined. Severity should also have been measured simultaneously or shortly after HbA1c measurements.

Our secondary outcomes were rates of local complications such as pancreatic cyst formation and pancreatic necrosis, systemic complications such as renal or respiratory failure, mortality rates, and any other detrimental outcome included in the studies. With these secondary outcomes in place, we aimed to obtain not only a clearer idea of how HbA1c levels affect AP severity, but also how they may alter the prognosis for patients with the disease.

Data Collection and Extraction

Literature searching and abstract screening were performed independently by two reviewers (AA and SL) using the ABSTRACKR tool (Center for Evidence Synthesis in Health, Brown University School of Public Health, Brown University, Providence, RI, USA) [19]. Full-text articles were then assessed against eligibility criteria, with disagreements resolved by discussion and consensus, and a third reviewer (BK) available if required.

Data extraction was conducted independently by the same two reviewers (AA and SL). Data were obtained from published reports or, where unavailable, by contacting study authors. If this process failed, the data were then obtained via other means, for example, by measuring the graphs included in the published material and then obtaining the relevant numeric data.

Quality Assessment

Once the finalised search was complete, a risk of bias assessment was conducted for each of the studies identified. For any randomised study, we opted to use the Cochrane Risk of Bias 2 (RoB 2) tool [20], and for any non-randomised study, the Newcastle-Ottawa scale (NOS) [21]. The assessments were carried out independently by two reviewers (AA and SL), with any disagreements being resolved either through discussion until a consensus was reached or through consultation with the third reviewer (BK). Any study deemed at too high a risk of bias was excluded. 

Data Synthesis and Meta-Analysis

A meta-analysis comparing the likelihood of developing severe AP (expressed as odds ratios (ORs)) in those with HbA1c <6.5% and HbA1c >6.5%, using those with mild severity as our control, was performed. For each analysis, a significance level of 95% was adopted, and a random effects model was used to account for the differences between each study. Methodological heterogeneity was assessed by closely analysing both the procedural aspects of the included studies as well as their population characteristics. Statistical heterogeneity was examined by calculating the I² value for each reported summary statistic. All of our meta-analyses were conducted using the Review Manager (RevMan) version 5.4, 2020 [22] software (The Cochrane Collaboration, London, UK).

For the secondary outcomes of our review, the data were pooled in a similar way to the severity measurements. For example, we displayed the overall proportion of those with organ failure in the group with an HbA1c <6.5%, while also calculating the OR for developing organ failure in this group compared to the group with an HbA1c level >6.5%. The same measures of heterogeneity were also implemented.

Results

**Figure 1:**
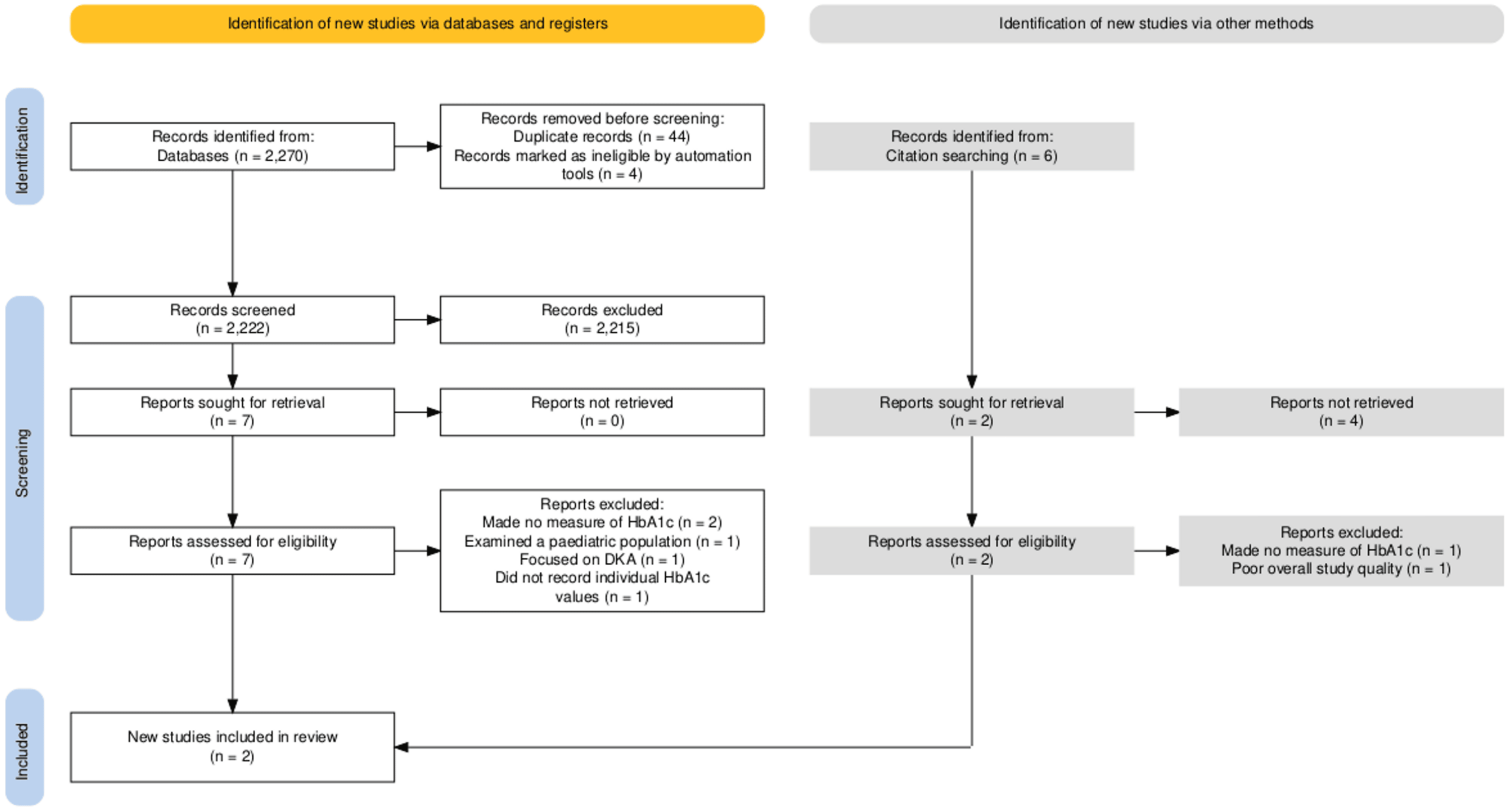
PRISMA diagram for review search process PRISMA: Preferred Reporting Items for Systematic Reviews and Meta-Analyses; DKA: diabetic ketoacidosis [23]

Figure 1 shows the yielded results of the search string. After removal of duplicates and non-English language studies, the total remaining was 2,222. After screening, seven reports were sought for retrieval, and, following detailed analysis, only two of the studies met the eligibility criteria. We then searched through the citations of the seven reports to find any other papers to include in our review, identifying six for assessment, none of which met our eligibility criteria. We also searched for any unpublished material or ongoing studies on the topic; however, nothing was uncovered.

Studies were excluded for several reasons, most commonly because they examined the relationship between diabetes and AP without measuring HbA1c [24-25]. Additional exclusions included one paediatric-only study [26], one assessing diabetic ketoacidosis and AP [27], and one examining hyperglycaemia without HbA1c measurement [28]. Following quality assessment, one further study was excluded due to poor methodological definition [29].

Another study by Zhao et al. [30] reported an association between elevated HbA1c and poorer AP outcomes but lacked sufficient data granularity for inclusion, and further clarification could not be obtained from the authors.

Ultimately, two studies met the inclusion criteria. Nagy et al. [31] primarily investigated the effects of glucose on AP but also reported baseline HbA1c and its association with disease severity. Han et al. [32] focused specifically on the relationship between HbA1c and AP severity. Both studies are outlined below.

Description of the Included Studies

Nagy et al. [31] conducted an international cohort study of 2,250 patients with AP, of whom 754 had available HbA1c data and were included in this review. Patients were stratified into five HbA1c categories (≤6.50%, 6.51%-7.00%, 7.01%-8.00%, 8.01%-9.00%, and ≥9.01%). The study reported AP severity (Atlanta Classification), organ failure, and mortality across groups. Most participants were from Hungary and other European countries; the sole non-European site in Japan contributed only two patients and was considered negligible.

Han et al. [32] performed a retrospective cohort study of 441 patients with AP, examining the association between HbA1c and disease severity and outcomes. Patients were classified as having normal HbA1c (<6.5%) or high HbA1c (≥6.5%), with the latter further subdivided into 6.5% ≤ HbA1c < 8%, 8% ≤ HbA1c < 10%, and ≥ 10%. Baseline characteristics and biochemical data were also collected. Participants were recruited from a single centre (The Affiliated Hospital of Yangzhou University) between January 2013 and December 2020. A summary of both studies is provided in Table 1.

**Table 1:** Summary of the included studies [31-32]

Author	Study design	Participants and location	HbA1c groups	Study methods	Acute Pancreatitis Severity Measure	Acute pancreatitis outcomes	Reported outcomes
Nagy et al. (2021)	Multi-centre international prospective cohort	Patients taken from multiple international centres, mainly Hungary and other European countries, N = 754, ≥18 years of age	1. ≤6.50%; 2. 6.51%–7.00%; 3. 7.01% – 8.00%; 4. 8.01% – 9.00%; 5. ≥9.01%	Participants were identified at admission and continually followed up throughout the duration of their stay; HbA1c could be collected at any time during hospitalisation	Atlanta Classification	Systemic/local complications, mortality rates, length of hospitalisation (LOH), maximal CRP	An observable but not statistically significant trend between raised HbA1c and pancreatitis severity and risk of local complications. Statistically significant trend between increased HbA1c and LOH and maximal CRP.
Han et al. (2023)	Retrospective cohort	Patients admitted to the Affiliated Hospital of Yangzhou University, China, N = 441, ≥18 years of age	1.<6.5%; 2. 6.5% ≤HbA1c <8%; 3. 8% ≤HbA1c <10%; 4. 10% ≤HbA1c.	Severity was determined retrospectively from patients admitted to the hospital with acute pancreatitis. HbA1c collected after admission. Local/systemic complication that occurred within 48 hours	Atlanta Classification	Systemic/ local complications Systemic inflammation response syndrome (SIRS) rates	Statistically significant trend of increasing HbA1c with pancreatitis severity, local complications, severe complications and SIRS rates

Population Characteristics

Nagy et al. [31] included an international but predominantly European set of participants, whilst Han et al. [32] collected data from one hospital in China. Each study featured more male than female patients, and the mean age was also similar, with Han et al. [32] featuring slightly younger participants on average compared to Nagy et al. [31]. The aetiology of AP among the included participants differed between the two studies; however, by far the most common cause in the study by Nagy et al. [31] was biliary disease, followed by alcohol, whereas hypertriglyceridemia was the leading cause in Han et al. [32], followed by biliary disease. Despite the differences between the two studies, we deemed that the profiles of each tested population were similar enough to be compared and meta-analysed.

Risk of Bias Assessment

Both studies were assessed for their risk of bias using the NOS, and, in each case, the risk was deemed to be low. Nagy et al. [31] was marked down for its failure to separate out patients with diabetes from each HbA1c group, unlike Han et al. [32], which made the distinction. The results of the assessment are summarised in Table 2.

**Table 2:** Newcastle–Ottawa Scale (NOS) risk of bias assessment Each star (★) represents one point awarded for meeting the NOS criteria in the corresponding domain. Higher total scores indicate greater methodological quality. [31-32]

Study	Representativeness of the exposed cohort	Selection of the non-exposed cohort	Ascertainment of exposure	Demonstration that the outcome of interest was not present at the start of the study	Comparability	Assessment of outcome	Follow-up length	Adequacy of follow-up
Nagy et al.	★	★	★			★	★	★
Han et al.	★	★	★		★	★	★	★

Outcomes

Both studies assessed AP severity using the Atlanta Classification and reported rates of organ failure and local complications, such as pancreatic necrosis. However, most outcome data were presented graphically rather than numerically. We contacted the study authors to obtain extractable data, but received no response; therefore, DigitizeIt software (Bormisoft, Braunschweig, Germany) [33] was used to extract values from published figures. These data were combined with available numerical results to generate tables and analyses. No data transformation was required, as outcomes were dichotomous and assessed consistently across both studies.

Each study also included some measures that were relevant to this review (such as mortality rates); although reported, the results could not be combined as they were not measured in both studies.

Severity Amongst the HbA1c Groups

Both studies assessed AP severity using the Revised Atlanta Classification. In Nagy et al. [31], rates of moderate AP increased from 20.6% in the HbA1c ≤6.50% group to 25.4% in the ≥9.01% group, though the highest rate occurred in the 8.01-9.00% group, indicating no clear dose-response relationship. Severe AP showed a similar pattern, rising from 5.9% (≤6.50%) to 9.7% (7.01-8.00%) before decreasing to 5.5% in the ≥9.01% group. Therefore, as expected, the p-value for trend was not significant (p=0.394).

In contrast, Han et al. [32] reported a stronger, dose-dependent association with increasing rates of moderate and severe AP at higher HbA1c levels. Severe AP rose from 5.3% in the <6.5% group to 26.7% in the ≥10% group, with a statistically significant trend (p<0.05).

Local Pancreatic Complications (Acute Peripancreatic Fluid Collections, Pseudocyst, Necrotic Collection, and Walled-Off Necrosis)

Each study measured the rate of local pancreatic complications as defined by the Atlanta Classification of AP severity, with Nagy et al. [31] providing extra detail by outlining the exact complication that arose (e.g., pancreatic pseudocyst). 

Nagy et al. [31] observed a trend of increasing rates of local complications with increasing HbA1c levels; however, it was found not to be statistically significant (p=0.122). The rate rose from 23.7% in the HbA1c ≤6.50% group to 30.9% in the ≥9.01% group. The rate, however, similar to the proportion of severe AP cases, was highest in the 8.01%-9% group, where the proportion of local complications was 36.3%. Han et al. [32] observed the same trend, with the rate of local complications increasing from 15.4% in the <6.5% group to 42.2% in the ≥10% HbA1c group. Unlike Nagy et al. [31], the trend they uncovered was statistically significant.

Systemic Complications

Both studies recorded the rates of organ failure amongst their participants; however, as with local complications, only Nagy et al. [31] described the type in greater detail.

Nagy et al. [31] found a similar trend to what they observed with the rates of severe AP, whereby the rates of organ failure at first increased from the ≤6.50% group to the 7.01%-8.00% group (8.04% to 12.5%), but then began to decrease in the two highest groups (9.03% and 5.32%, respectively). This trend was seen amongst all the organ failure subcategories that Nagy et al. [31] provided further detail on (respiratory, heart and renal failure) and was deemed not to be statistically significant (p=0.959).

Han et al. [32], on the other hand, found a less uncertain relationship, with the rates of organ failure increasing from 6.16% in the <6.5% group to 50% in the ≥10% HbA1c group.

Other Reported Outcomes

Outside the previously mentioned findings, there were some reported outcomes relevant to this review; however, they were not measured in both studies, and thus, the results could not be pooled. In the study performed by Nagy et al. [31], the researchers also commented on the varying mortality rates in each group, as well as the average length of hospitalisation and the maximal CRP levels. Han et al. [32], on the other hand, recorded the rates of systemic inflammatory response syndrome (SIRS) amongst their groups.

For mortality, Nagy et al. [31] found no real trend amongst the groups, as mortality rates were highest in the 6.51-7.00% group (7.16%) and lowest in the 7.01-8.00% group (3.14%). Conversely, they found that HbA1c was directly associated with the length of hospitalisation and maximal CRP. Han et al. [32] noted how the SIRS rate increased with HbA1c, with the proportion rising from 13.8% in the <6.5% group to 32.1% in the ≥10% HbA1c group. 

In addition, Nagy et al. [31] performed a binary logistic regression along with a receiver operating characteristic curve (ROC) analysis to determine the predictive capability of HbA1c. The binary logistic regression they performed did not show HbA1c to be an independent predictor of mortality (OR = 1.211 (95% CI: 0.859-1.646), p = 0.241) or severity (OR = 1.028 (95% CI: 0.768-1.332), p = 0.843). Their ROC analysis showed that HbA1c fails to predict mortality (area under the curve (AUC) = 0.545) and poorly predicts the severity of AP (AUC = 0.601).

Meta-Analysis of Available Results

Since each study used the Atlanta Classification both to measure the severity of AP and define local and systemic complications, the results were able to be combined without modification. Han et al. [32] did not provide a breakdown of the HbA1c group sizes; instead only described the number of participants with an HbA1c level lower than or equal to 6.5% and those with a level higher. Both studies also categorised their HbA1c groups differently, and so, without the individual HbA1c levels for each participant, meta-analysis of more specific HbA1c levels was impossible. We therefore conducted meta-analyses for the following reported outcomes: AP severity, organ failure and local complications.

AP Severity

**Figure 2:**
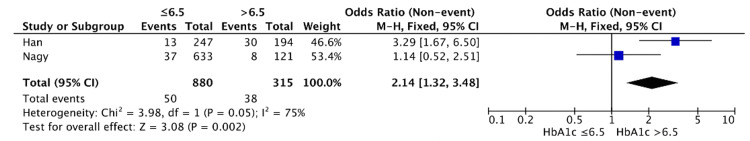
Pooled OR for the risk of severe acute pancreatitis by HbA1c Heterogeneity: Chi² = 3.98, df = 1, P = 0.05; I² = 75%; Test for overall effect: Z = 3.08, P = 0.002. CI: confidence interval; M–H: Mantel–Haenszel method; df: degrees of freedom [31-32]

We performed a meta-analysis by calculating the OR for developing severe AP for those with an HbA1c>6.5%, compared to those with an HbA1c >6.5% using patients with mild AP as our comparator. The results are displayed in Figure 2.

The calculated OR for both studies share the same direction of effect, and so both show an increased odds of severe AP with elevated HbA1c. The OR for Nagy et al. [31] showed less of an effect than in Han et al. [32]; however, this is most likely explained by the small number of events in the HbA1c >6.5% group (n=8). This fact also probably accounts for the high calculated I^2^ value of 75%.

The overall OR of 2.14 (95% CI 1.32-3.48) suggests that the odds of developing severe AP are 2.14 times greater for patients with an HbA1c >6.5% compared to those with an HbA1c ≤6.5%.

Organ Failure

**Figure 3:**
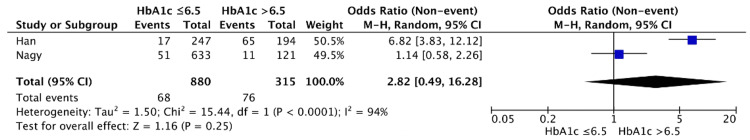
Pooled OR for the risk of organ failure by HbA1c Heterogeneity: τ² = 1.50; Chi² = 15.44, df = 1, P < 0.0001; I² = 94%; Test for overall effect: Z = 1.16, P = 0.25. CI: confidence interval; M–H: Mantel–Haenszel method; df: degrees of freedom [31-32]

The likelihood of developing organ failure with differing levels of HbA1c is found in Figure 3.

The ORs for both studies suggest an increased likelihood for developing organ failure when HbA1c is greater than 6.5; however, the ratio is larger in the study conducted by Han et al. [32] (6.82 compared to 1.14). This is most likely due to the small number of events (n=11) in Nagy et al.’s [31] study, which will also have contributed to the high I² value of 94%.

Whilst the pooled OR of 2.82 does show an increased likelihood of developing organ failure in AP patients when HbA1c levels are raised, the CI has a large range of 0.49 to 16.28, highlighting the uncertainty of the true effect calculated by the meta-analysis.

Local Complications

**Figure 4:**
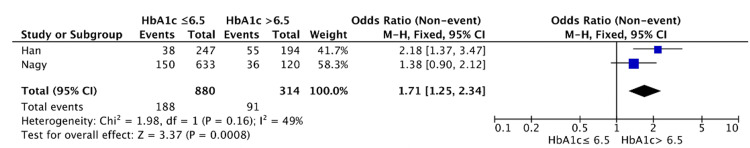
Pooled OR for the risk of local complications by HbA1c Heterogeneity: Chi² = 1.98, df = 1, P = 0.16; I² = 49%; Test for overall effect: Z = 3.37, P = 0.0008. CI: confidence interval; M–H: Mantel–Haenszel method; df: degrees of freedom References: [31–32]

Our final meta-analysis examined the likelihood of developing local complications in patients with AP with HbA1c levels greater than 6.5% to those less than or equal to 6.5%. The results are displayed below in Figure 4.

The calculated ORs for both studies show that the odds of developing local complications are increased with raised HbA1c levels. The number of events in Nagy et al’s [31] was much more comparable than for previous outcomes (n=36), which in turn has resulted in both narrow calculated CIs for our pooled OR and a smaller I^2^ value of 49%. 

The pooled OR of 1.71 shows an increased odds of developing local complications when HbA1c is >6.5%. Compared to the results of our previous meta-analyses, the results presented in Figure 4 can be accepted with the greatest degree of confidence, but still must be taken with caution due to the small number of studies drawn from. 

Discussion

Summary of Results

Our systematic review and meta-analyses found supportive evidence that higher HbA1c levels are associated with increased odds of developing severe AP, as well as local and systemic complications. While the strength of evidence varied by outcome, with the strongest being for local complications, each meta-analysis demonstrated a positive association between rising HbA1c levels and poorer AP outcomes.

Given the limited research available, the mechanisms underlying these associations remain speculative. One proposed explanation is supported by a previous review suggesting that elevated serum CRP levels in diabetes may lead to pancreatic islet β-cell dysfunction and apoptosis [34], which could in turn exacerbate AP [35]. In keeping with this, Nagy et al. [31] reported a direct association between increasing HbA1c levels and maximal CRP values, suggesting a potential causal pathway. An alternative explanation is that elevated HbA1c reflects a state of chronic hyperglycaemia [36], with persistently raised glucose levels, rather than glycated haemoglobin itself, driving the observed effects. Chronic hyperglycaemia increases intracellular reactive oxygen species (ROS) [37], which can promote pancreatic inflammation via mitochondrial dysfunction and cell death [38]. ROS are also implicated in microvascular endothelial dysfunction [39], which is particularly relevant given the pancreas’s susceptibility to ischaemic injury and may further exacerbate AP [40].

These findings have important clinical implications for the management of AP. Firstly, they suggest that HbA1c may serve as a prognostic indicator, consistent with findings from Nagy et al. and Han et al. [31-32]. Secondly, AP prognosis is strongly influenced by the development of organ failure and local complications [41]. If elevated HbA1c contributes to these outcomes, improved glycaemic control may offer prognostic benefit. This is particularly relevant given that AP itself is associated with the subsequent development of diabetes, especially in cases involving pancreatic necrosis [42]. Maintaining good HbA1c control may therefore reduce diabetes risk both directly through improved glycaemic regulation [43] and indirectly by reducing AP-related pancreatic damage that predisposes to diabetes [10].

Quality of the Evidence

A major limitation across both included studies was the handling of diabetes as a potential confounder in the relationship between HbA1c and AP. This issue was more pronounced in the study by Nagy et al. [31], where participants with and without diabetes were not analysed separately. Although Han et al. [32] did report differences between participants with HbA1c ≤6.5% and >6.5% according to diabetes status, this distinction was not reflected in the graphical data from which most values for our meta-analyses were extracted.

Another limitation of the Nagy et al. study [31] was the marked imbalance between HbA1c groups, with 83.85% of participants (633 of 754) having HbA1c ≤6.50%. This reduced the statistical power to detect significant differences between groups [44] and contributed to the high heterogeneity and wider confidence intervals observed in our pooled odds ratios [45]. In contrast, Han et al. [32] included a more even distribution of participants (247 with HbA1c ≤6.5% and 194 with HbA1c >6.5%). However, while Nagy et al. [31] provided a detailed breakdown of participant numbers across HbA1c categories above 6.5%, Han et al. [32] did not. As a result, the precise contribution of individual HbA1c subgroups above 6.5% to the overall findings remains unclear, as the allocation of these 194 participants was not disclosed.

Strengths of the Review

To our knowledge, this is the first review to examine the available evidence on the relationship between HbA1c and AP. We sought to maximise study inclusion by searching multiple databases, screening reference lists, and reviewing unpublished material where available. We also attempted to obtain missing or unclear data directly from study authors.

In addition, we explicitly addressed the limitations of both the included studies and our own meta-analyses. We incorporated statistical measures to assess evidence quality and employed several strategies to minimise bias, including individual risk-of-bias assessments.

Limitations of the Review

Despite efforts to conduct a comprehensive search, non-English language studies were excluded. Although unlikely, this raises the possibility that relevant evidence may have been missed [46]. The overall quality of included studies could also have been higher, potentially affecting the reliability and validity of the findings [47]. These issues stemmed not only from the inherent limitations of the study designs but also from methodological weaknesses, particularly in addressing confounding variables [48].

Finally, only two studies met the inclusion criteria, limiting the strength of conclusions that can be drawn. This contributed to variable heterogeneity, wide confidence intervals, and restriction of analyses to only two HbA1c groups [45]. Inclusion of additional high-quality studies would be required to address these limitations and strengthen the evidence base.

## Conclusions

Considering the limited body of evidence regarding the association between HbA1c and pancreatitis severity, it is difficult to draw any definitive conclusions at this point. Despite this, alongside the flaws of the studies included, the results do highlight a possible association between increasing HbA1c levels and AP severity, as well as the development of systemic and local complications, which is worth further study.

At present, no implications can be made to current clinical practice until more definitive evidence is available. This would ideally be a large prospective cohort study in diabetic and non-diabetic populations with efforts to reduce the effect of confounding, particularly due to comorbid diseases associated with diabetes.

## Appendices

Appendix A 

**Table 3:** Search string for the systematic review

Search string for systematic review	
S1	Haemoglobin A1c
S2	Hemoglobin A1c
S3	HbA1c
S4	Glycosylated haemoglobin A1c
S5	Glycosylated hemoglobin A1c
S6	Glycated haemoglobin A1c
S7	Glycated hemoglobin A1c
S8	Glycosylated haemoglobin A1c
S9	Glycated hemoglobin A1c
S10	S1 OR S2 OR S3 or S4 OR S5 OR S6 OR S7 OR S8 OR S9
S11	Pancreatitis
S12	S10 AND S11
